# Correspondence about the article "Left ventricular systolic longitudinal strain in mechanically ventilated patients in the intensive care unit: assessment of global and chamber reproducibility"—author’s reply

**DOI:** 10.1186/s40635-025-00789-x

**Published:** 2025-09-25

**Authors:** Matías Pécora, Piero Pastorini, Roberto Farolini, Gastón Burghi, F. Javier Hurtado

**Affiliations:** 1 Unidad Académica de Fisiopatología, Hospital de Clínicas, Facultad de Medicina, Universidad de la República, Montevideo, Uruguay; 2 Laboratorio de Exploración Funcional Respiratoria, Centro de Tratamiento Intensivo, Hospital de Clínicas, Facultad de Medicina, Montevideo, Uruguay; 3 Centro de Tratamiento Intensivo, Hospital de Clínicas, Facultad de Medicina, Universidad de la República, Montevideo, Uruguay; 4 Unidad de Cuidados Intensivos – Hospital Español, Administración de los Servicios de Salud del Estado (ASSE), Montevideo, Uruguay

We thank Dr. Aishwarya Raparthi and Dr. Sharanya Kumar Bavurothu for their valuable comments [1] on our recently published article [2].

This study evaluated the feasibility and reproducibility of left ventricular systolic strain (SL-S) and ejection fraction (LVEF) in mechanically ventilated ICU patients, who are the main population but also pose challenges for this technique. In addition to limited knowledge about this population's characteristics, especially the proportion on mechanical ventilation, in prior ICU feasibility-reproducibility studies. We included a diverse cohort that reflects the inherent clinical heterogeneity in daily practice. The aim was not to evaluate or categorize SL-S and LVEF into different hemodynamic phenotypes, and assess diagnostic thresholds and clinical outcomes of these parameters. An important preliminary step before examining diagnostic thresholds or prognostic relationships is to determine whether these parameters are feasible and reproducible. Understanding how much of a parameter’s variability is due to measurement error rather than true physiological change is key to avoiding both overdiagnosis and missed diagnoses, which could lead to inappropriate management decisions. Throughout the manuscript, we discuss that although SL-S and automated LVEF appear to be reproducible and feasible, further research is necessary to confirm these findings in larger populations and specific groups, such as those with sepsis and reduced LVEF. We agree to include those mentioned by the authors, such as load dependence, vasopressor support, or different hemodynamic phenotypes.

We completely agree with the authors that atrial fibrillation (AF) is a common arrhythmia in critically ill patients. The decision to exclude was based on the well-known technical limitations and unstandardized measurement of strain analysis in this population [3]. These limitations highlight the need for standard techniques to improve strain accuracy in AF patients.

We recognize the small sample size and lack of prospective justification for the required sample size. This is a common limitation in reproducibility research. We recognize the importance of following the Guidelines for Reporting Reliability and Agreement Studies (GRRAS) [4]. Retrospectively, the sample size estimation for SL-S reproducibility was evaluated (Supplementary material; Appendix 1; Appendix 2). The sample sizes estimated ranged from 8 to 46 subjects. The number of patients in our study aligns with or is slightly below the suggested range approaches. Nonetheless, the observed intraclass correlation coefficient (ICC) values were consistent with, or even higher than, those previously reported in the literature, and their confidence intervals (CI) remained narrow, suggesting high measurement stability and low variability, particularly when automatic tools are used to minimize subjectivity. These findings support the reliability of our results, although larger future studies are necessary to confirm these observations. A post hoc power analysis was performed (supplementary material; Appendix 3), indicating acceptable statistical power. These results provide statistical support for our findings, despite the single-center design and small sample size. We acknowledge that post hoc power analyses do not replace prospective sample size estimation and are mainly descriptive, reflecting the observed effect size and precision rather than providing independent evidence of study adequacy.

We acknowledge the author’s comment regarding the width of the CI in per-chamber ICC estimates; however, this is seen in non-critical reproducibility studies. Automatic SL-S demonstrated narrower CI across per-chamber views compared to the manual SL-S, suggesting greater measurement stability and potential advantages in ICU settings, where image quality may present significant challenges. Furthermore, the CI width for automatic SL-S is narrower than reported for global SL-S ICU settings [2].

It is relevant to recognize that no universally accepted ICC threshold defines excellent reproducibility. In our study, we used a cutoff of 0.90, though others have considered values ≥ 0.80 to indicate excellent agreement.

We appreciate the concern regarding the assumption of normality underlying the Bland–Altman analysis (see the normality test in the supplementary material, Appendix 4). Limits of agreement (LoA) in Bland–Altman analysis tend to be higher in per-chamber analysis; in fact, this is observed in non-critical reproducibility studies. It is recognized that LoA derived from small samples may underestimate measurement variability. To address this, we adhered to current reporting standards by conducting a Bland–Altman [5] (Supplementary material; Appendix 5). These findings suggest limited measurement error and acceptable agreement, even under conservative assumptions. In conclusion, although a small sample size remains a limitation, the use of  CI around the LoA and transparent reporting reduces the risk of overestimating precision.

We acknowledge the limitation of not performing a sensitivity analysis to assess the relationship between the reproducibility of SL-S or LVEF and vasopressor dosage, mean arterial pressure (MAP), ventilator settings, or preload dependence. To partially address this concern, we conducted an exploratory post hoc correlation analysis with MAP and systolic pressure, which revealed no significant associations. This result is likely underpowered due to the narrow range of values and limited sample size. A post hoc exploratory reproducibility analysis comparing patients who received vasopressors with those who did not showed no differences (Supplementary Material, Appendix 6).

Considering the level of training in echocardiography, MP was trained in transthoracic echocardiography from 2016 to 2019 (level III; *“COCATS 4”*) by two expert echocardiographers who were involved in MP's master's thesis (Supplementary material; Appendix 7). RF and PP are course level II trainees. All observers followed a defined protocol for SL-S measurements consistent with current guidelines detailed in our article and supplementary material [2].

Our original protocol involved assessing right ventricular longitudinal strain, which was only possible in 43% of the cohort. As a result, we did not conduct a reproducibility analysis.

We acknowledge the feasibility limitations of the two-chamber view; this issue highlights a potential area for future research. While current SL-S global assessments require integration of all three apical views, future studies could explore the diagnostic and prognostic value of SL-S derived from a single apical view, such as the four-chamber view. In clinical contexts, where myocardial dysfunction is typically diffuse, a single-view SL-S may offer a feasible and informative alternative when global acquisition is not possible. This approach could enhance the practicality of SL-S in ICU settings and facilitate broader clinical adoption.

## Supplementary Information

Additional file 1.

## Data Availability

The datasets used and/or analysed during the current study are available from the corresponding author on reasonable request.
