# Supplementary material for: Correspondence about the article "Left ventricular systolic longitudinal strain in mechanically ventilated patients in the intensive care unit: assessment of global and chamber reproducibility"—author’s reply
Source: Intensive Care Med Exp. 2025 Sep 25;13:97. doi: 10.1186/s40635-025-00789-x (PMC12463789; doi:10.1186/s40635-025-00789-x)
Supplement: Supplementary file 1 — Additional file 1. [file 40635_2025_789_MOESM1_ESM.docx]

Supplementary material

Content

[Appendix 1 2](#_Toc204252407)

[1A) Sample size calculation with the confidence interval approach (https://wnarifin.github.io/ssc/ssicc.html). 2](#_Toc204252408)

[1B) Sample size calculation for the testing approach on software R [3, 4]. 3](#_Toc204252409)

[1C) Sample size calculation through the interactive R Shiny application with all the methods discussed by Mondal et al. 4](#_Toc204252410)

[Appendix 2 7](#_Toc204252411)

[Appendix 3 8](#_Toc204252412)

[Appendix 3A 9](#_Toc204252413)

[Appendix 3B 9](#_Toc204252414)

[Appendix 3C 10](#_Toc204252415)

[Appendix 4 11](#_Toc204252416)

[Appendix 5 13](#_Toc204252417)

[Appendix 6 15](#_Toc204252418)

[Appendix 7 16](#_Toc204252419)

[Bibliography 17](#_Toc204252420)

## Appendix 1

Sample size determination for intraclass correlation coefficient (ICC) of intraobserver reproducibility, using the three established approaches: the confidence interval width approach, the assurance probability approach, and the testing approach [1]. For the confidence interval approach, we applied Bonett’s formula [2] (appendix 1A). The testing approach was determined from an available code in the software R [3] (Appendix 1B) [1, 4, 5]. Mondal et al. [1] developed a Shiny app that integrates all approaches for determining the sample size (Appendix 1C).

### 1A) Sample size calculation with the confidence interval approach (https://wnarifin.github.io/ssc/ssicc.html).


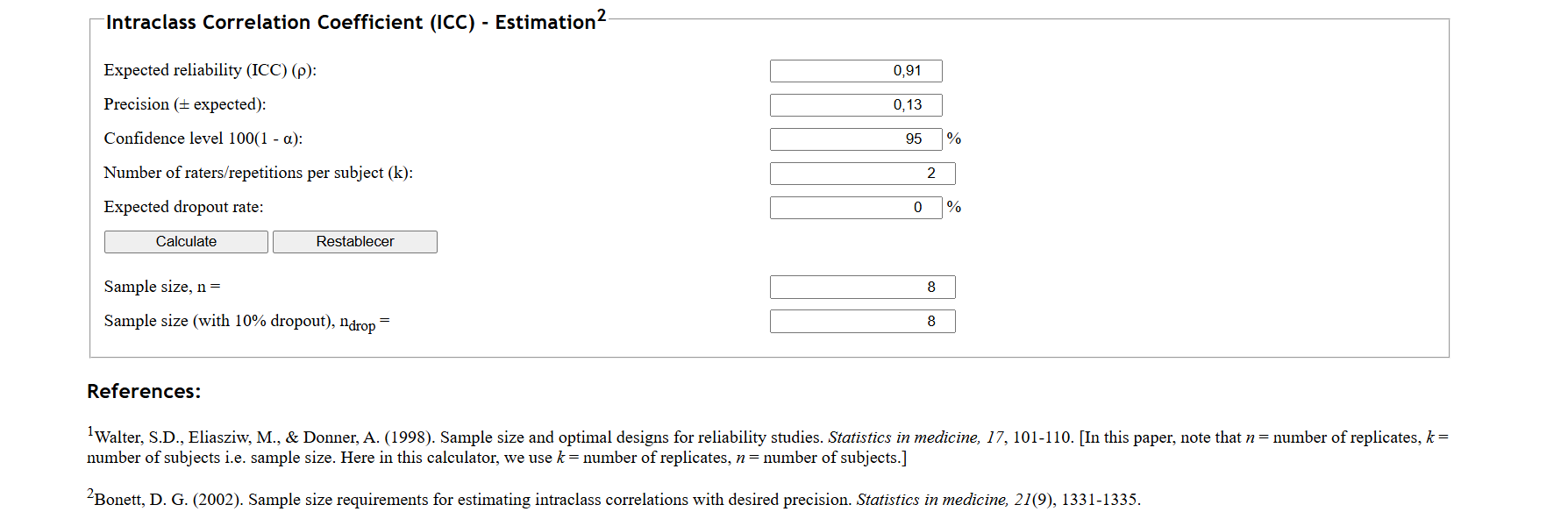


References:

Expected reliability was determined based on the average intraobserver reproducibility intraclass Correlation Coefficient (ICC) of the studies represented in Table 4 (Pecora et al. [1]) in the Intensive Care Unit context (except for the current study). Average ICC=0.91.

Precision was determined based on the average range of the confidence interval (CI) of the studies represented in Table 4 (Pecora et al. [1]) (except for the current study). Average range CI =0.26 (±0.13). Due to the large CI reported, we set a more stretched range of 0.15 (±0.075). See figure below. Sample size estimated (red box).


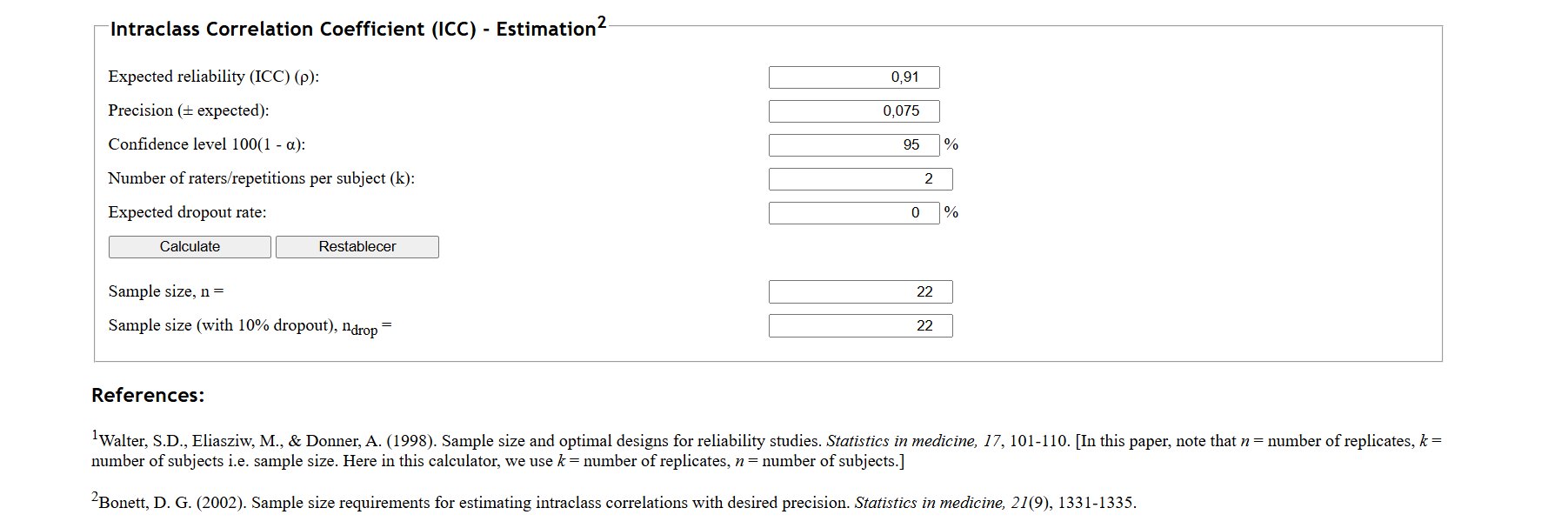


### 1B) Sample size calculation for the testing approach on software R [3, 4].

Sample size calculation for the testing approach, normalized Searle method (*ZF𝜌),* with different null hypotheses (p0) values (0.7; 0.75; 0.80).


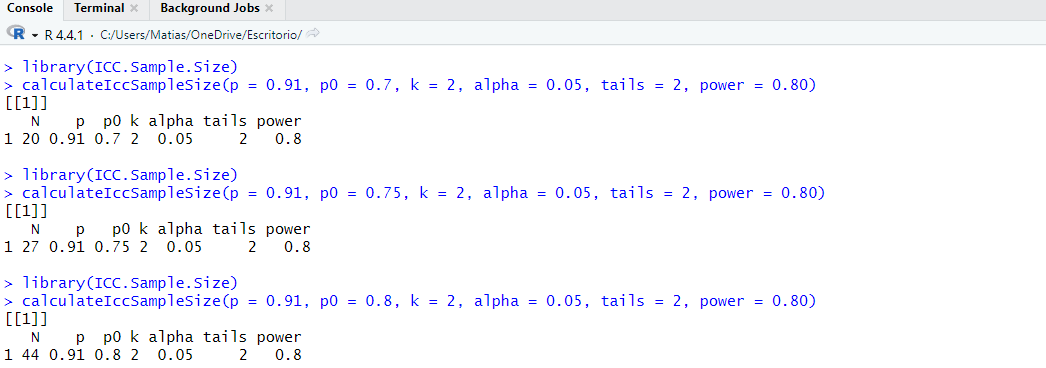


References:

p: hypothesized value of p, based on previous data. p0: null hypothesis value of p. k: number of ratings of each subject. Alpha: desired alpha for hypothesis testing. Tails: number of tails for hypothesis test. Power: desired power of the hypothesis test. N: sample size estimated (red box).

1C) Sample size calculation through the interactive R Shiny application with all the methods discussed by Mondal et al. [1] (https://github.com/DiproMondal/sample-size-ICCGithub).

**Sample size estimation by the confidence interval approach.**


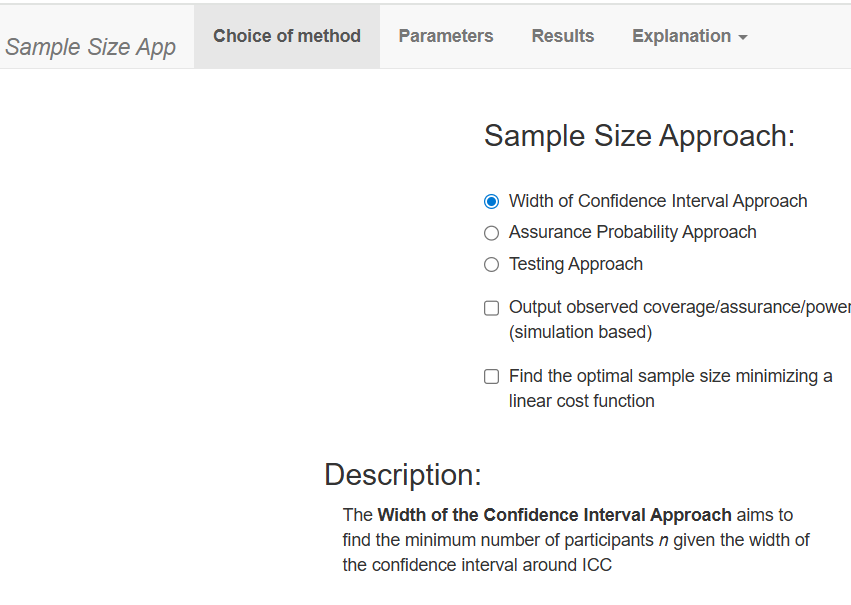

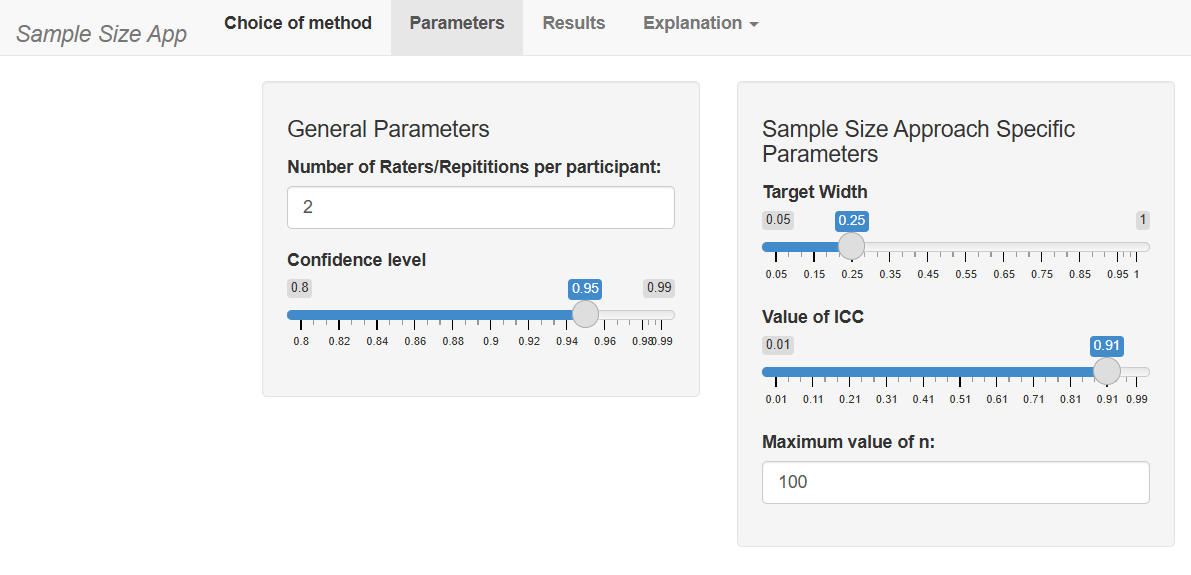

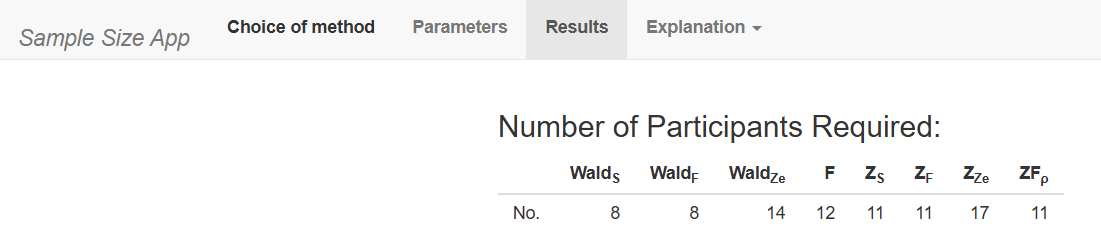


**References:**

Value of ICC: Expected reliability was determined based on the average intraobserver reproducibility intraclass Correlation Coefficient (ICC) of the studies represented in Table 4 (Pecora et al [6]) in the Intensive Care Unit context ( with the exception of the current study). Average ICC=0.91.

Target Width: Precision was determined based on the average range of confidence interval (CI) of the studies represented in Table 4 (Pecora et al [6]) in the Intensive Care Unit context ( with the exception of the current study). Average range CI =0.26 (±0.13). Due to the large CI reported, we set a more stretched range of 0.15 (±0.075). See the Image below (green box).

No (Red box): sample size estimated with eight confidence interval methods. The sample sizes obtained using the different confidence interval methods are relatively close. In all the approaches, the Searle method (F) ( purple circle) provided a sample size with good statistical properties as well as normalized Searle method (ZFp) (raters=2) (orange circle) as described by Mondal et al. [1].


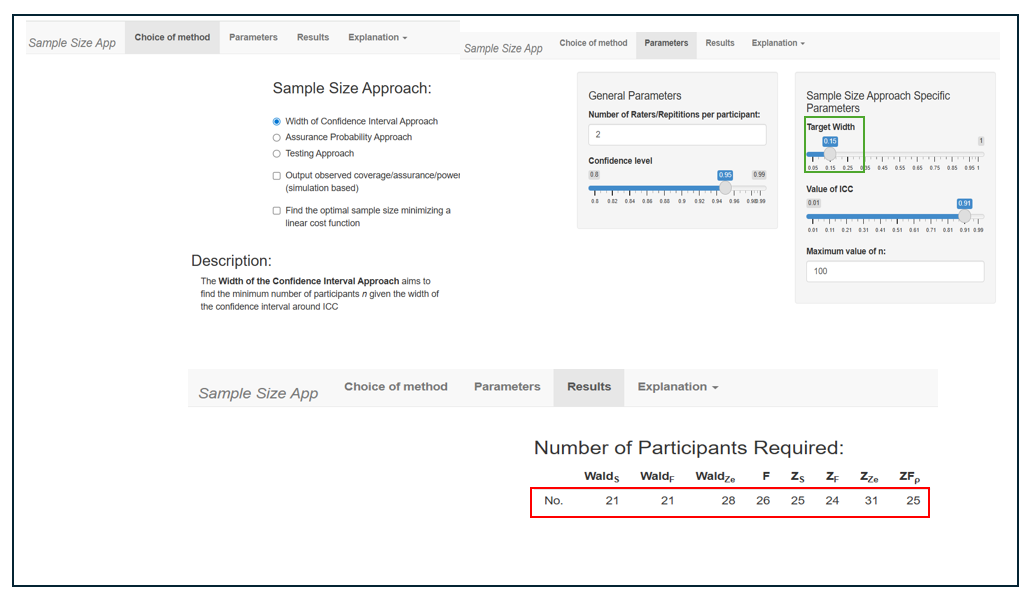


References:

Target Width: selected a more stretched range of 0.15 (±0.075) (green box).

**Sample size estimation by testing approach.**


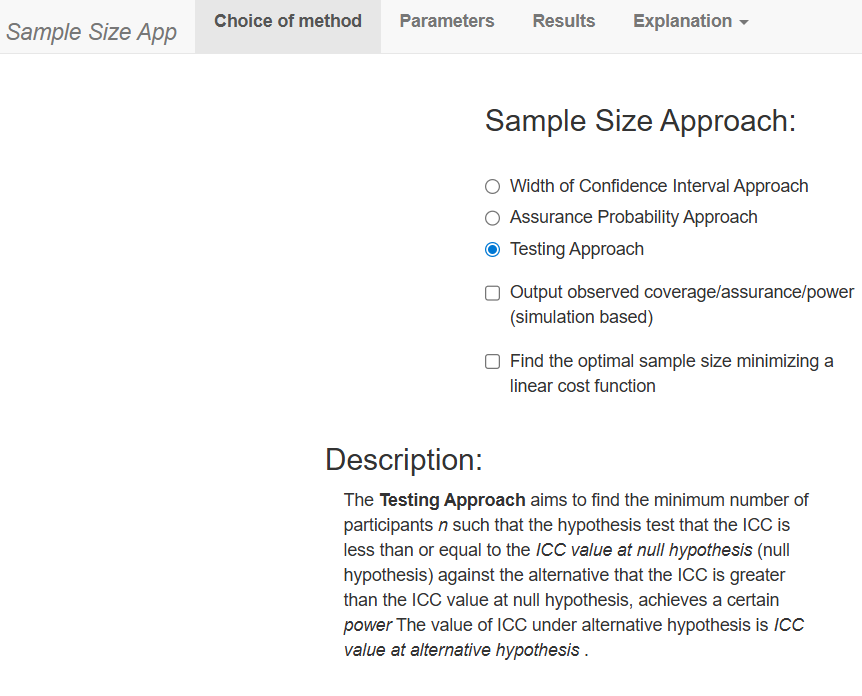

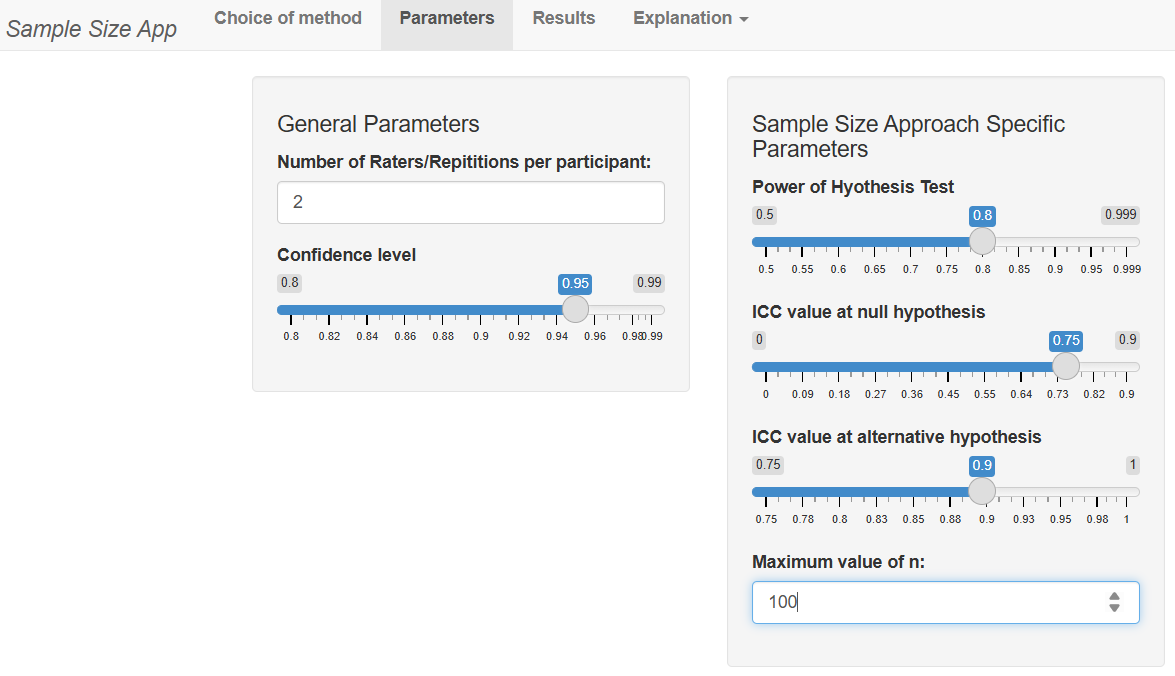

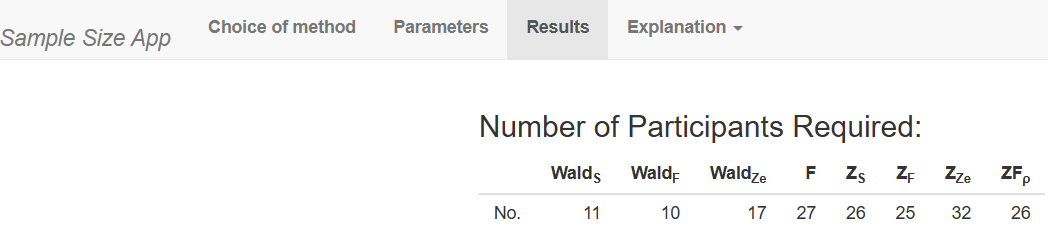


References:

k: number of ratings of each subject.

ICC value at null Hypothesis: minimum ICC acceptable value, 0.75, considering the minimum threshold for good reproducibility, 0.80, considering a higher exigence threshold (see figure below; green box).

ICC value at the alternative Hypothesis: ICC value to demonstrate in the study. In this case, we select based on the average of previous studies (Pecora et al[6] ; table 4)

Power of the hypothesis test: desired power of the hypothesis test.

No (Red box): sample size estimated with eight confidence interval methods. The sample sizes obtained using the different confidence interval methods are relatively close. In all the approaches, the Searle method (F) (purple circle) and ZFp (orange circle) provide a sample size with good statistical properties as described by Mondal et al [1].


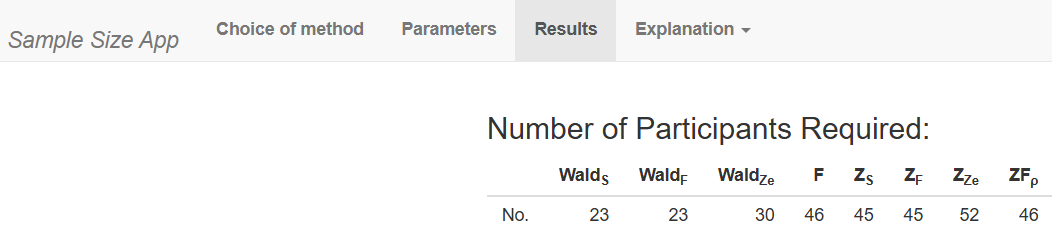

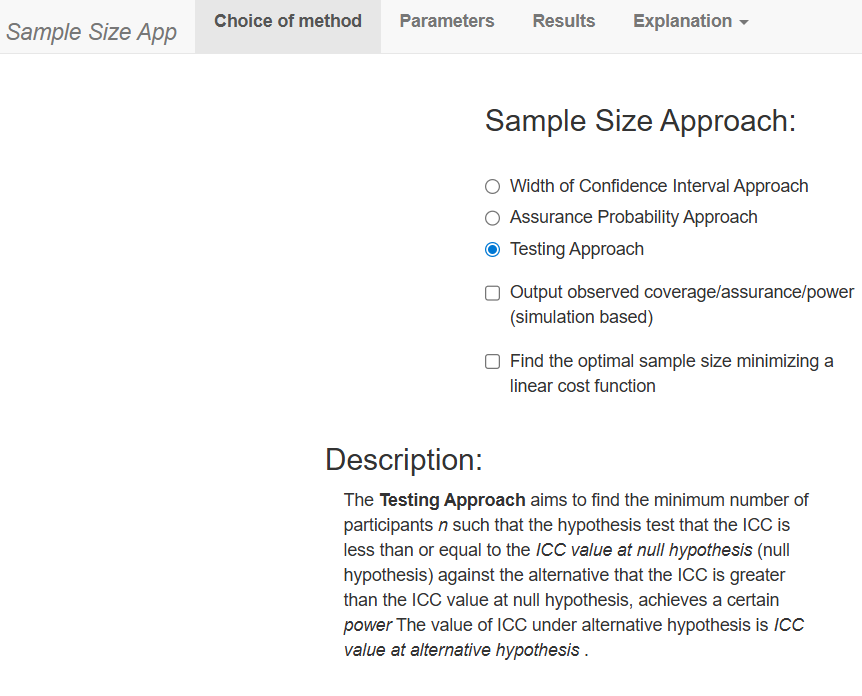

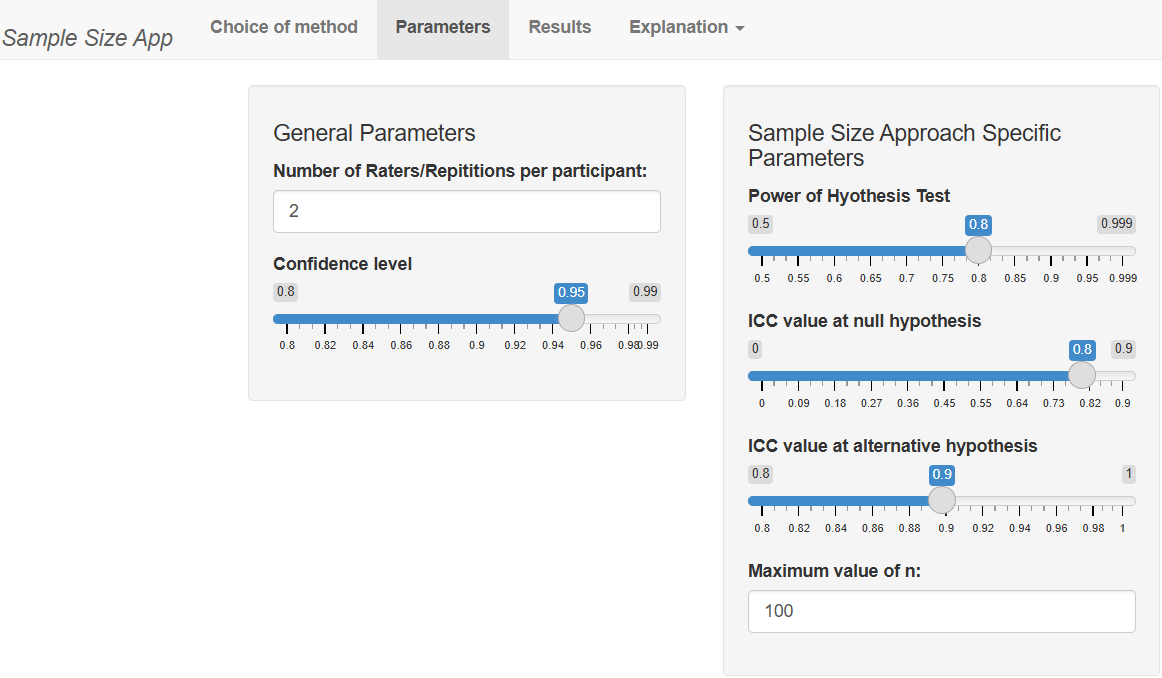


References:

ICC value at null Hypothesis: minimum 0.80, considering a higher exigence threshold (green box).

## Appendix 2

Sample size estimation for interobserver reproducibility was performed using the simulation-based method implemented in the Shiny app described by Mondal et al. [7] (See image below; Appendix 2). The estimated sample size was n = 17 subjects, which yields an average 95% confidence interval of approximately ±0.084, corresponding to a total width of 0.168 for the ICC. Notably, the actual CI observed in our study for the interobserver ICC of SL-S was even narrower, with a total width of 0.06, suggesting lower variability and higher agreement than expected under conservative planning assumptions.

Sample size estimation, two-way ANOVA using R code (shiny::runGitHub('DiproMondal/sample-size-ICCagreement-2wayANOVA')) or Shiny app (https://dipro.shinyapps.io/sample-size-icc/ app) created by Monda et al [7].


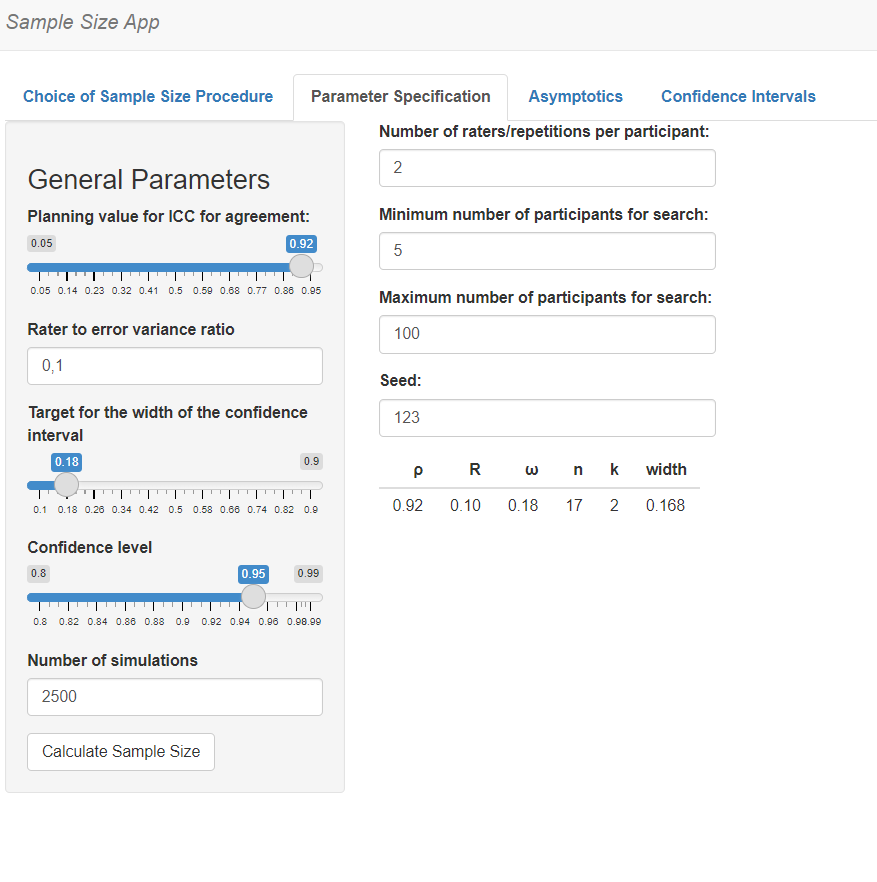

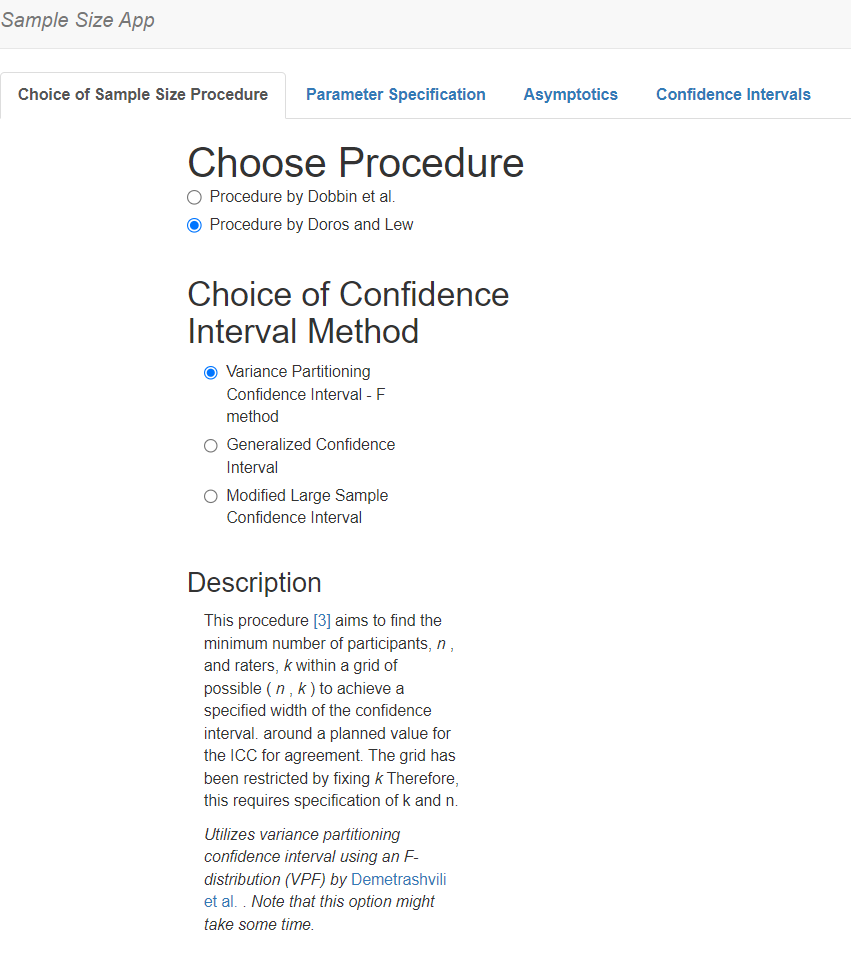


References.

Model Two-Way: interobserver reproducibility (2 or more raters per subject). Procedure of Doros and Lew.

Approach estimation: 𝑉𝑃𝐹 (Variance Partitioning F distribution).

Planning value for ICC for agreement (ρ): 0.92. Expected or target ICC based on the average of ICC reported in previous studies (Pecora et al. [6]) .

Rater to error to variance ratio: ratio of variance between evaluators and error. Variability attributed to evaluators. Estimated R: 0.1

k (repetitions): Number of raters (2 observers) per subject.

Target for the width of the confidence interval (ω): Desired total width of the 95% confidence interval for ICC, expected width (ω = 0.18) was selected based on the average width of ICC intervals reported in previous studies (Pecora et al. [6])

Confidence Level: 0.95.

The estimated sample size was n = 17 subjects (red box), which yields an average 95% confidence interval of approximately ± 0.084, corresponding to a total width of 0.168 for the ICC (green box).

## Appendix 3

A post hoc power analysis was performed on software R based on the method proposed by Zou et al. [4] to assess the statistical strength of the reproducibility findings. This approach enables the evaluation of whether the observed ICC and sample size were sufficient to detect a level of agreement above a predefined threshold. Although post hoc power does not replace prospective sample size planning, it offers valuable insight into whether the study’s design was adequate given the observed effect size [8].

In the intraobserver reproducibility analysis (Appendix 3A) of SL-S, the observed ICC was 0.97 (95% CI: 0.92–0.98). Using 20 patients and two raters, the null hypothesis ICC (p₀) was set at 0.89, based on previously published intraobserver SL-S reproducibility studies in ICU patients (Pecora et al [6]; table 4), which reported ICCs ranging from 0.88 to 0.97. This threshold reflects the lower boundary of what is considered acceptable reproducibility in similar critically ill populations and aligns with the CI limits found in the literature. The statistical power to detect a significant difference from a clinically acceptable ICC of 0.89 was 83.2% (appendix 3A). These results suggest that the study was sufficiently powered to detect a level of agreement above the predefined threshold, reinforcing the validity of the intraobserver reproducibility findings despite the modest sample size. For the interobserver reproducibility analysis, the same threshold (p₀ = 0.89) was applied. This value was derived from published interobserver ICCs for SL-S, ranging from 0.91 to 0.94, with lower confidence bounds between 0.73 and 0.88. Therefore, this threshold represents the minimum acceptable agreement level in similar ICU contexts and applying it to both analyses maintains consistency in power assessment.

### Appendix 3A

Post-hoc calculation of the statistical power of the observed ICC for SL-S on software R [4]


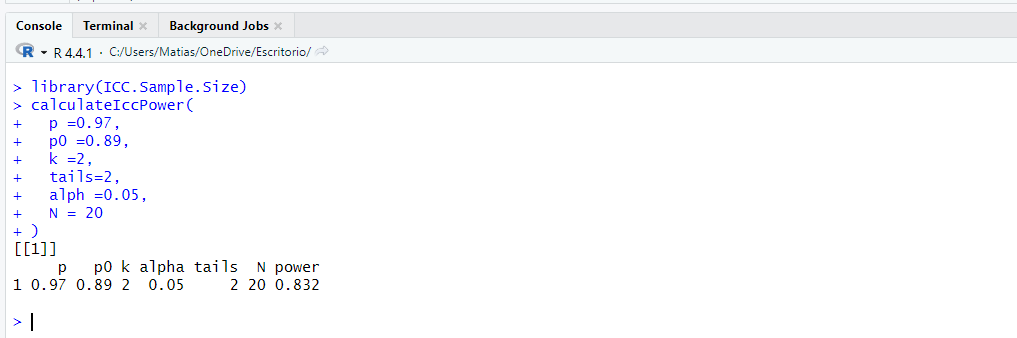


References:

Observed intraobserver ICC current study = (p) 0.97; Null hypothesis ICC= (p₀) 0.89; this value was selected considering the ICC values reported in ICU context ( Pecora et al[6], Table 4). Number of raters (k)=2; Significance level (α)=0.05; Sample size (N)= 20 patients; Poer: Statistical power estimated = 0.832 (83.2%) (Red box). This exceeds the conventional 80% threshold and indicates that the study had sufficient ability to identify differences even within a high ICC range.

### Appendix 3B

Exploratory reproducibility analysis by the ICC comparing SL-S automatic interobserver reproducibility with SL-S manual interobserver reproducibility involving three raters. The reproducibility remained excellent, with an ICC of 0.97 (95% CI: 0.93–0.99). A post hoc power analysis was conducted using this observed ICC value of 0.97, with three raters and 20 subjects (See image below). The power to detect a significant difference from a predefined threshold was 92.7% (Appendix 3C). These results strengthen the methodological support for our findings, despite the exploratory and single-center design. [8].


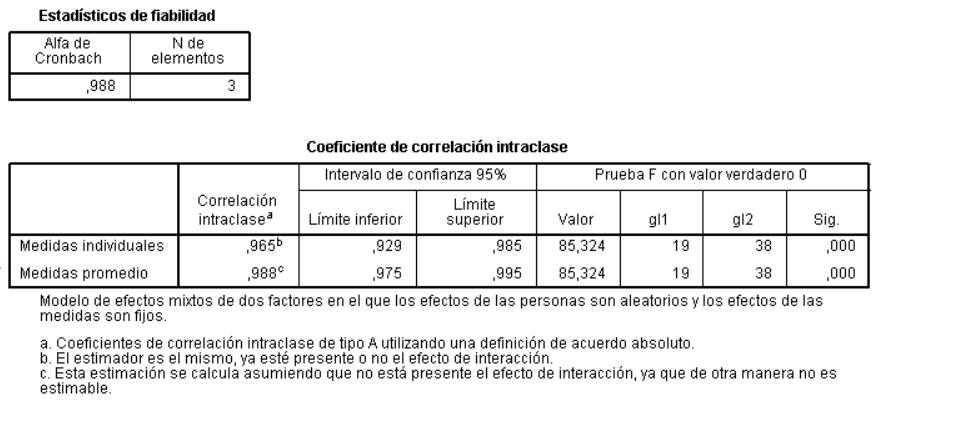


References: ICC value and its confidence intervals, with individual measurements (red box)

### Appendix 3C

Post-hoc calculation of the statistical power of the automatic ICC for SL-S (3 raters) on software R.


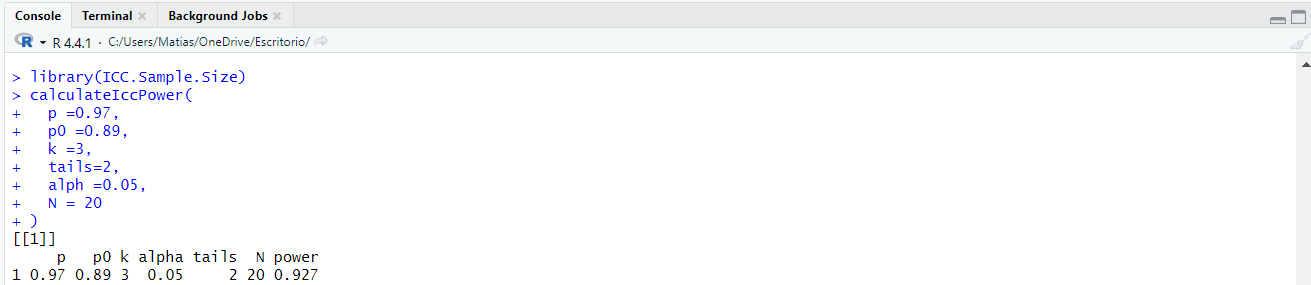


References:

Observed ICC current study = (p) 0.97; Null hypothesis ICC= (p₀) 0.89; Number of raters (k)=3; Significance level (α)=0.05; Sample size (N)= 20 patients; Statistical power (red box)= 0.927 (92.7 %).

## Appendix 4

Normality assessment through the Shapiro-Wilk test. SPSS v19®

| **Pruebas de normalidad** | | | | | | |
| --- | --- | --- | --- | --- | --- | --- |
|  | Kolmogorov-Smirnov^a^ | | | Shapiro-Wilk | | |
|  | Estadístico | gl | Sig. | Estadístico | gl | Sig. |
| DIF_AFI_SLS_INTRA | ,118 | 20 | ,200^*^ | ,975 | 20 | ,849 |
| a. Corrección de la significación de Lilliefors  *. Este es un límite inferior de la significación verdadera. | | | | | | |

Abbreviations: DIF_AFI_SLS_INTRA: Differences between measurements for automated SL-S intraobserver.

| **Pruebas de normalidad** | | | | | | |
| --- | --- | --- | --- | --- | --- | --- |
|  | Kolmogorov-Smirnov^a^ | | | Shapiro-Wilk | | |
|  | Estadístico | gl | Sig. | Estadístico | gl | Sig. |
| DIF_AFI_SLS_INTER | ,162 | 20 | ,180 | ,941 | 20 | ,252 |
| a. Corrección de la significación de Lilliefors | | | | | | |

Abbreviations: DIF_AFI_SLS_INTRA: Differences between measurements for automated SL-S interobserver.

| **Pruebas de normalidad** | | | | | | |
| --- | --- | --- | --- | --- | --- | --- |
|  | Kolmogorov-Smirnov^a^ | | | Shapiro-Wilk | | |
|  | Estadístico | gl | Sig. | Estadístico | gl | Sig. |
| DIF_SLS_INTER | ,106 | 20 | ,200^*^ | ,951 | 20 | ,388 |
| a. Corrección de la significación de Lilliefors  *. Este es un límite inferior de la significación verdadera. | | | | | | |

Abbreviations: DIF_SLS_INTER: Differences between measurements for manual SL-S interobserver.

| \| **Pruebas de normalidad** \| \| \| \| \| \| \| \| --- \| --- \| --- \| --- \| --- \| --- \| --- \| \|  \| Kolmogorov-Smirnov^a^ \| \| \| Shapiro-Wilk \| \| \| \| Estadístico \| gl \| Sig. \| Estadístico \| gl \| Sig. \| \| DIF_AFI_3C_INTRA \| ,098 \| 20 \| ,200^*^ \| ,968 \| 20 \| ,710 \| \| a. Corrección de la significación de Lilliefors  *. Este es un límite inferior de la significación verdadera. \| \| \| \| \| \| \|   Abbreviations: DIF_AFI_3C_INTRA: Differences between measurements for automated 3 chamber SL-S intraobserver.   \| **Pruebas de normalidad** \| \| \| \| \| \| \| \| --- \| --- \| --- \| --- \| --- \| --- \| --- \| \|  \| Kolmogorov-Smirnov^a^ \| \| \| Shapiro-Wilk \| \| \| \| Estadístico \| gl \| Sig. \| Estadístico \| gl \| Sig. \| \| DIF_SLS_3C_INTRA \| ,109 \| 20 \| ,200^*^ \| ,939 \| 20 \| ,234 \| \| a. Corrección de la significación de Lilliefors  *. Este es un límite inferior de la significación verdadera. \| \| \| \| \| \| \|   Abbreviations: DIF_SLS_3C_INTRA: Differences between measurements for automated 3 chamber SL-S intraobserver.   \| **Pruebas de normalidad** \| \| \| \| \| \| \| \| --- \| --- \| --- \| --- \| --- \| --- \| --- \| \|  \| Kolmogorov-Smirnov^a^ \| \| \| Shapiro-Wilk \| \| \| \| Estadístico \| gl \| Sig. \| Estadístico \| gl \| Sig. \| \| DIF_SLS_3C_INTER \| ,155 \| 20 \| ,200^*^ \| ,916 \| 20 \| ,085 \| \| a. Corrección de la significación de Lilliefors  *. Este es un límite inferior de la significación verdadera. \| \| \| \| \| \| \|   Abbreviations: DIF_SLS_3C_INTER: Differences between measurements for manual 3 chamber SL-S interobserver. |
| --- | --- | --- | --- | --- | --- | --- | --- | --- | --- | --- | --- | --- | --- | --- | --- | --- | --- | --- | --- | --- | --- | --- | --- | --- | --- | --- | --- | --- | --- | --- | --- | --- | --- | --- | --- | --- | --- | --- | --- | --- | --- | --- | --- | --- | --- | --- | --- | --- | --- | --- | --- | --- | --- | --- | --- | --- | --- | --- | --- | --- | --- | --- | --- | --- | --- | --- | --- | --- | --- | --- | --- | --- | --- | --- | --- | --- | --- | --- | --- | --- | --- | --- | --- | --- | --- | --- | --- | --- | --- | --- | --- | --- | --- | --- | --- | --- | --- | --- | --- | --- | --- | --- |

## Appendix 5

Bland-Altman graph illustrating interobserver reproducibility by Automatic SL-S with 95% confidence intervals (CI) for the mean and Limits of Agreement (LoA), which are rarely reported in echocardiography reproducibility studies [4, 14]. This approach provides a more accurate estimation of uncertainty, particularly relevant when working with modest sample sizes, which are common in reproducibility studies [9–11]. Including confidence bounds helps address the risk of underestimating variability in conventional Bland–Altman analysis.


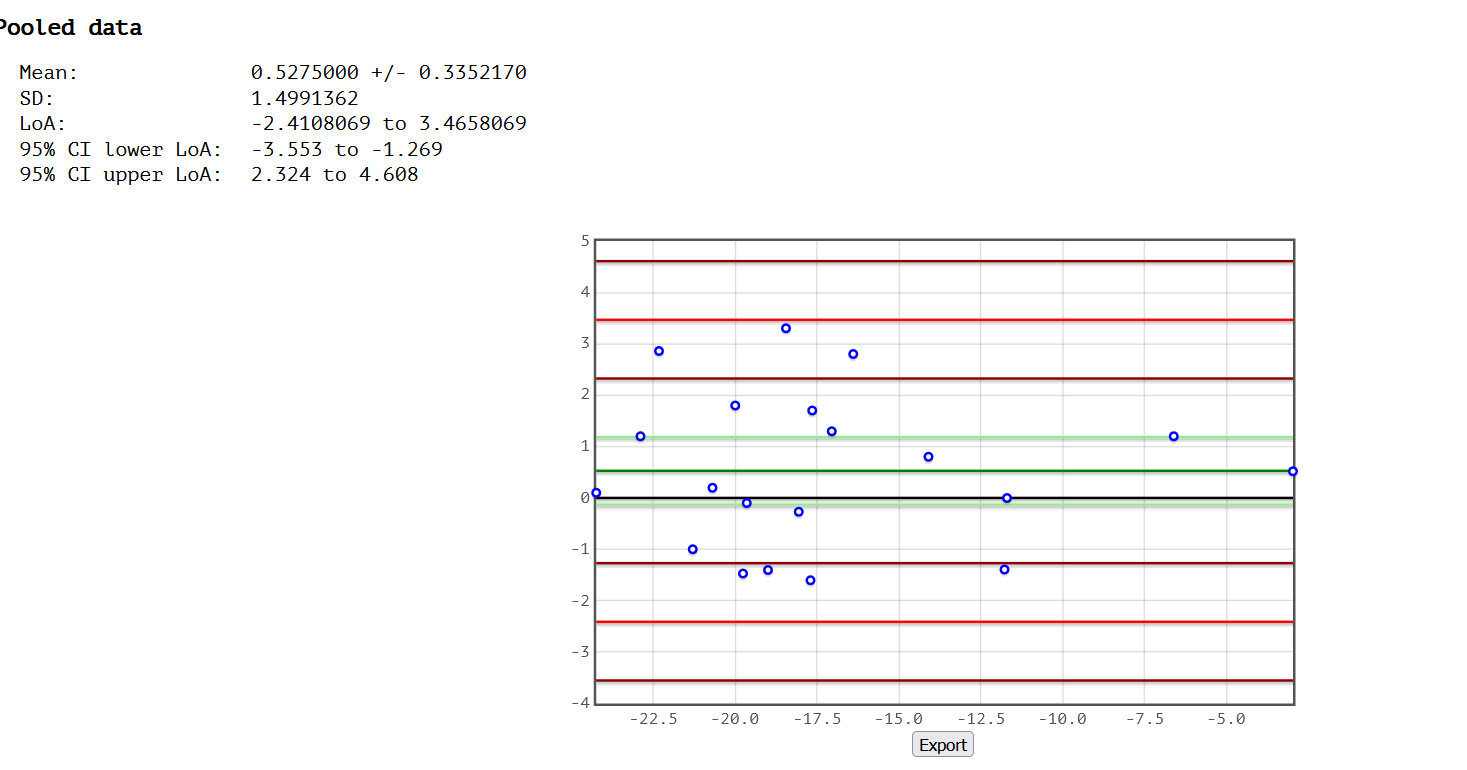


Interobserver difference (%)

Mean Automatic SL-S (%)

References:

Interobserver reproducibility of the automated SL-S yielded: A low bias of 0.53 ± 0.33; LoA ranging from –2.41 to 3.47; And their corresponding 95% CIs: –3.55 to –1.27 for the lower LoA; 2.32 to 4.61 for the upper LoA.

Bland-Altman graph illustrating interobserver reproducibility by manual SL-S with confidence intervals (CI) 95% for mean and Limits of agreement (LoA).


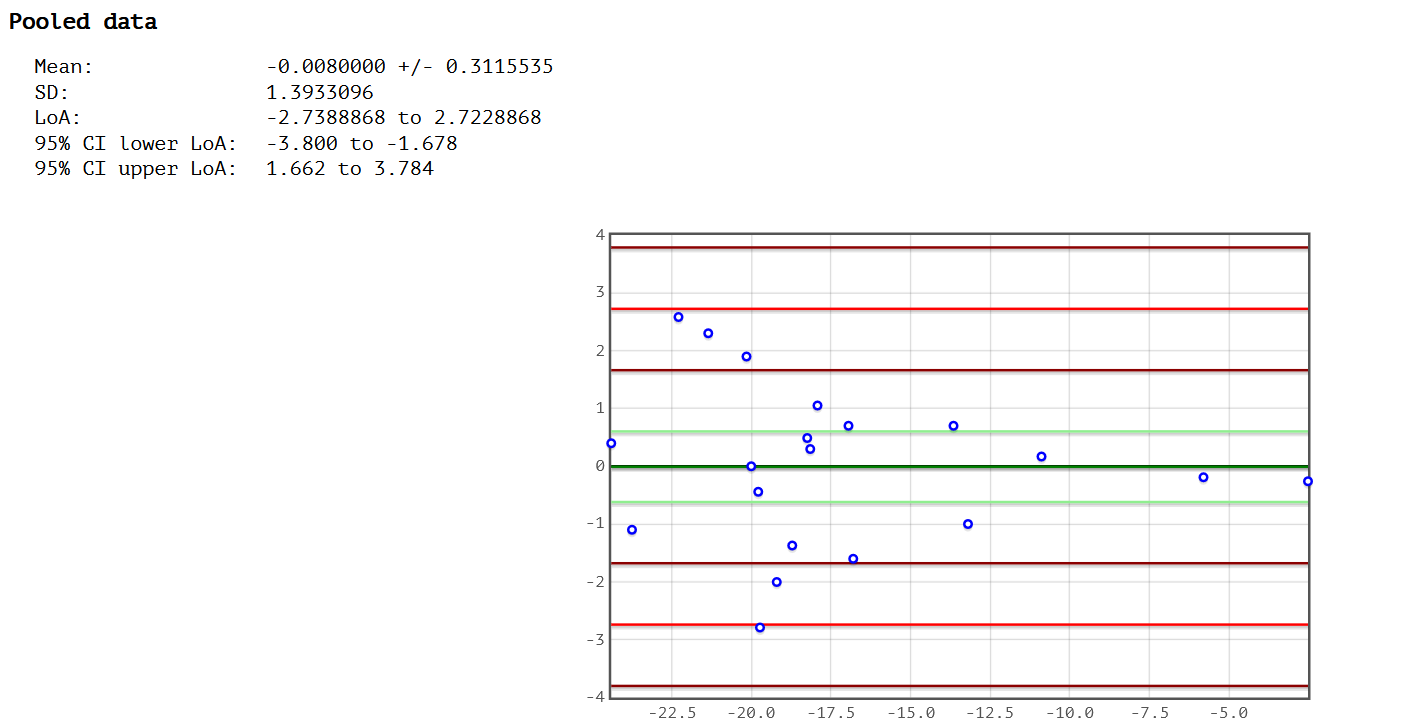


Interobserver difference (%)

Mean Manual SL-S (%)

References:

Interobserver reproducibility manual SL-S measurements, the bias was negligible (–0.008 ± 0.31), with LoA from –2.74 to 2.72 and 95% CIs from –3.80 to –1.68 (lower) and 1.66 to 3.78 (upper)

## Appendix 6

Reproducibility analysis regarding the use of vasopressors.

| Intraclass correlation coefficient (95% CI) | | | | |
| --- | --- | --- | --- | --- |
|  | **Under vasopressor (N=8)** | | **Without vasopressor (N=12)** | |
|  | **Intraobserver** | **Interobserver** | **Intraobserver** | **Interobserver** |
| Manual SL-S | 0.97 (0.51-0.99) * | 0.98 (0.90-0.99) * | 0.98 (0.93-0.99) * | 0.97 (0.89-0.99) * |
| Automatic SL-S | 0.97 (0.79-0.99) * | 0.96 (0.83-0.99) * | 0.98 (0.94-0.99) * | 0.96 (0.88-0.99) * |
| Manual LVEF | 0.84 (0.25-0.97) | 0.74 (0.07-0.95) | 0.81 (0.46-0.94) * | 0.82 (0.18-0.95) * |
| Auto-LVEF | 0.96 (0.83-0.99) * | 0.94 (0.75-0.99) * | 0.93 (0.78-0.98) * | 0.93 (0.74-0.98) * |
| Intra and interobserver reproducibility is represented by the intraclass correlation coefficient and 95% confidence interval (95% CI). **P<0.001*  SL-S: LV global systolic longitudinal ventricular strain. Manual LVEF: left ventricular ejection fraction using the modified biplanar Simpson’s method. Auto-LVEF: left ventricular ejection fraction using automatic GE® software. | | | | |

| Bland-Altman Reproducibility | | | | |
| --- | --- | --- | --- | --- |
|  | **Under vasopressor (N=8)** | | **Without vasopressor (N=12)** | |
|  | **Intraobserver** | **Interobserver** | **Intraobserver** | **Interobserver** |
| Manual SL-S | 0.24 (1.03); (-0.78 – 3.27) | -0.51 (1.22); (-2.90 – 1.87) | 0.31 (1.06); (-1.76 – 2.39) | -0.33 (1.45); (-2.51 – 3.16) |
| Automatic SL-S | 0.99 (1.29); (-1.50 – 3.50) | 0.65 (1.7); (-2.7 – 4.00) | -0.09 (0.97); (-1.98 – 1.79) | 0.45 (1.41); (-2.32 – 3.21) |
| Manual LVEF | -6.87 (7.79); (-22.14 – 8.40) | 10.37 (7.4); (-4.13 – 24.89) | -3.75 (7.36); (-18.28 – 10.68) | 5.67 (5.68); (-5.46 – 16.80) |
| Auto-LVEF | 0.75 (4.92); (-10.39 – 8.89) | 1.00 (5.37); (-9.52 – 11.52) | -1.10 (4.64); (-10.78 – 8.01) | 2.25 (3.74); (-5.08 – 9.58) |
| Bland–Altman analysis represents intra- and interobserver reproducibility: the mean difference and its standard deviation (SD) are represented, as are the upper and lower limits of agreement (lower–upper)  SL-S: LV global systolic longitudinal ventricular strain, Manual LVEF left ventricular ejection fraction using the modified biplanar  Simpson’s method, Auto-LVEF left ventricular ejection fraction using automatic GE® software | | | | |

## Appendix 7

URL: https://hdl.handle.net/20.500.12008/45497

Pécora, M. (2022.). Relación entre la rigidez aórtica y la deformación longitudinal del ventrículo izquierdo en una cohorte poblacional. Tesis de maestría. Universidad de la República (Uruguay). Facultad de Medicina. PROINBIO.

## Bibliography

1. Mondal D, Vanbelle S, Cassese A, Candel MJ (2024) Review of sample size determination methods for the intraclass correlation coefficient in the one-way analysis of variance model. Stat Methods Med Res 33:532–553. https://doi.org/10.1177/09622802231224657

2. Bonett DG (2002) Sample size requirements for estimating intraclass correlations with desired precision. Statistics in Medicine 21:1331–1335. https://doi.org/10.1002/sim.1108

3. R Core Team (2020) R: A language and environment for statistical computing. https://www.R-project.org

4. Alasdair Rathbone , Saurabh Shaw, Dinesh Kumbhare (2015) ICC.Sample.Size: Calculation of Sample Size and Power for ICC. https://CRAN.R-project.org/package=ICC.Sample.Size. Accessed 9 Jul 2025

5. Zou GY (2012) Sample size formulas for estimating intraclass correlation coefficients with precision and assurance. Statistics in Medicine 31:3972–3981. https://doi.org/10.1002/sim.5466

6. Pécora M, Pastorini P, Farolini R, et al (2025) Left ventricular systolic longitudinal strain in mechanically ventilated patients in the intensive care unit: assessment of global and chamber reproducibility. ICMx 13:. https://doi.org/10.1186/s40635-025-00770-8

7. Mondal D, Candel MJJM, Cassese A, Vanbelle S (2025) Confidence Intervals and Sample Size for the ICC in Two‐Way ANOVA Models. Statistics in Medicine 44:. https://doi.org/10.1002/sim.70106

8. Lakens D (2022) Sample Size Justification. Collabra: Psychology 8:. https://doi.org/10.1525/collabra.33267

9. Han O, Tan HW, Julious S, et al (2022) A descriptive study of samples sizes used in agreement studies published in the PubMed repository. BMC Med Res Methodol 22:. https://doi.org/10.1186/s12874-022-01723-5

10. Carkeet A (2015) Exact Parametric Confidence Intervals for Bland-Altman Limits of Agreement. Optometry and Vision Science 92:e71–e80. https://doi.org/10.1097/opx.0000000000000513

11. Abu-Arafeh A, Jordan H, Drummond G (2016) Reporting of method comparison studies: a review of advice, an assessment of current practice, and specific suggestions for future reports. British Journal of Anaesthesia 117:569–575. https://doi.org/10.1093/bja/aew320
